# Higher Ed as a Leveler: How Early Life and Higher Education Shape Resilience and Health in Adulthood

**DOI:** 10.1093/geroni/igaa057.1273

**Published:** 2020-12-16

**Authors:** Tyler Bruefach, Dawn Carr

**Affiliations:** Florida State University, Tallahassee, Florida, United States

## Abstract

Growing evidence shows that individuals who have high levels of psychological resilience maintain higher levels of physical and psychological health in later life. Individuals cultivate psychological resilience over the life course, yet little research has explored its mechanistic effects on health during midlife. One source of resilience may be formal education, which is a well-established determinant of health in adulthood. Resilience might be one reason for this robust association, as education helps individuals develop greater psychological resources in adulthood. On the other hand, having a college degree also increases access to other health-promoting resources that can be leveraged over the life course, such as better-paying and higher-quality jobs. Using data drawn from the National Longitudinal Study of Adolescent to Adult Health (Add Health), the current paper examines: 1) how early-life factors shape psychological resilience in early adulthood (24-32 years); and 2) the effects of early adulthood resilience on the association between education and health in mid-life (36-44 years). Results show that psychological resilience and college education have significant direct effects on health in midlife, net of health in early adulthood. However, first-generation college graduates cultivate more psychological resilience from their educational attainment than do those with college-educated parents. That is, higher education serves as a leveler for health gaps in midlife for those with fewer resources available in early life by bolstering resilience. These results provide important insights about how early life factors play an important role in shaping successful aging processes.

